# COVID-19 and thrombotic risk in patients with active cancer: a matched cohort study

**DOI:** 10.1093/jncics/pkag110

**Published:** 2026-10-12

**Authors:** Jérôme Doyen, Alexandre Lacour, Renaud Schiappa, Eva Seutin, Amanda Garrido, Tanguy Pace Loscos, Denis Doyen

**Affiliations:** Department of Radiation Oncology, Antoine Lacassagne Cancer Center, Côte d’Azur University, Nice, France; Department of Gastroenterology, Centre Hospitalier Universitaire de Nice, Nice, France; Department of Epidemiology, Biostatistics and Health Data, Centre Antoine-Lacassagne, Côte d’Azur University, Nice, France; Department of Epidemiology, Biostatistics and Health Data, Centre Antoine-Lacassagne, Côte d’Azur University, Nice, France; ICBMS—Institut de Chimie et de Biochimie Moléculaires et Supramoléculaires, Equipe COSSBA UMR 5246, Lyon, France; Department of Epidemiology, Biostatistics and Health Data, Centre Antoine-Lacassagne, Côte d’Azur University, Nice, France; Unité d’Explorations Fonctionnelles, Centre Hospitalier Universitaire de Nice, Côte d'Azur University, Nice, France; LP2M, Laboratoire de Physiomédecine Moléculaire, CNRS, Côte d’Azur University, Nice, France

## Abstract

Coronavirus disease 2019 (COVID-19) has been associated with an increased thrombotic risk, potentially exacerbated in patients with active cancer. We conducted a retrospective cohort study including 119 cancer patients with COVID-19 and 214 matched prepandemic controls. Patients with COVID-19 had poorer performance status and were more frequently treated with palliative intent. After a median follow-up of 32.7 months, overall thrombotic event rates did not differ between groups. However, after adjustment for clinically relevant covariates and accounting for death as a competing event, COVID-19 status and palliative treatment intent remained independently associated with a higher cumulative incidence of thrombotic events. In conclusion, COVID-19 may increase thrombotic risk in patients with cancer and occurs more frequently in patients with poorer clinical status. These findings are exploratory and require confirmation in larger prospective studies.

Coronavirus disease 2019 (COVID-19), caused by severe acute respiratory syndrome coronavirus 2 (SARS-CoV-2), is associated with a systemic inflammatory response that contributes to endothelial dysfunction and hypercoagulability.^1^ In the general population, COVID-19 has been linked to an increased risk of arterial and venous thrombotic events, with estimated absolute risks of approximately 0.5% and 0.25%, respectively, after 1.4 million COVID-19 diagnoses.^2^ Cancer is independently associated with a prothrombotic state due to tumor-related mechanisms.^3^ Consequently, patients with active malignancy may be at particularly high risk of thrombotic complications when infected with SARS-CoV-2, which may in turn contribute to excess mortality.

Meta-analyses have consistently identified cancer as an independent risk factor for mortality among patients with COVID-19.^4^^,^^5^ However, these studies did not evaluate whether thrombotic events contributed to the increased mortality risk, nor did they compare outcomes with a control population of patients with cancer but without COVID-19.

Thus, it remains uncertain whether COVID-19 confers an additional thrombotic risk beyond that associated with malignancy itself. Addressing this question requires direct comparison between patients with cancer with and without COVID-19. We therefore conducted a retrospective matched cohort study to evaluate the incidence of thrombotic events and overall survival (OS) in these populations.

We performed a retrospective single-center cohort study including adult patients (≥18 years) with histologically confirmed cancer receiving active treatment and a diagnosis of COVID-19 confirmed by reverse-transcriptase polymerase chain reaction testing. Patients were identified at a comprehensive cancer center between March 2020 and December 2022, corresponding to the period during which systematic COVID-19 screening was implemented for hospital admission. The duration of follow-up was extended to the maximum available, as thrombotic events may occur several months after SARS-CoV-2 infection.^6^^,^^7^ Patients with life-threatening emergencies (ie, acute respiratory distress or hemodynamic instability) were not included. The Khorana score was calculated at hospital admission using baseline clinical and laboratory parameters. For control patients, the score was calculated at the first documented medical assessment associated with an available blood test. Prophylactic anticoagulation mainly consisted of subcutaneous enoxaparin at 4000 IU once daily (6000 IU once daily in patients with a body mass index [BMI] > 30 kg m^−^^2^). Therapeutic anticoagulation mainly consisted of weight-adjusted low-molecular-weight heparin (predominantly tinzaparin at 175 anti-Xa IU kg^−1^ once daily), according to clinical indication. Overall, 62 patients received antiplatelet therapy and 102 received anticoagulant therapy; among the latter, 99 received anticoagulation with prophylactic intent and 3 with therapeutic intent. A total of 119 patients were included in the COVID-19 cohort.

A matched control cohort of patients with cancer without COVID-19 (*n* = 214) was selected from the prepandemic period (January 2011-October 2017) to ensure absence of SARS-CoV-2 infection. Patients were identified using a data warehouse and matched according to ICD-10 codes (tumor site and metastatic status), age at diagnosis (10-year range), and sex. Patients were matched using a hierarchical exact-matching approach. A pre-COVID-19 control group was preferred over a contemporaneous cohort because of the known limitations in sensitivity of early SARS-CoV-2 diagnostic tests, particularly in asymptomatic or early infection.^8^ The patient identification, matching, and selection process is summarized in the STROBE-style flow diagram (Figure S1). In accordance with the Declaration of Helsinki, the study was approved by the French National Health Committee for Data Protection (CNIL) (registration number: MR-004, no. F20230419153727). All patients and/or their families received written information about the study and provided consent for the anonymous use of patient data for research purposes.

The primary endpoint was the occurrence of venous or arterial thrombotic events. Secondary endpoints included OS and identification of predictive factors for thrombosis. Thrombotic events were defined as arterial events (including myocardial infarction, ischemic stroke, and other arterial thromboses) or venous events (including pulmonary embolism, deep vein thrombosis, portal vein thrombosis, and intracranial venous thrombosis), all confirmed using standard imaging techniques.

Overall survival was defined as the interval from COVID-19 diagnosis to death from any cause. For the control cohort, the reference date was defined as the date of the first documented medical assessment associated with an available blood test, as laboratory values were required to calculate the Khorana score.

Potential predictors of thrombosis included age, sex, cardiovascular risk factors, Eastern Cooperative Oncology Group (ECOG) performance status, hospitalization status, COVID-19–related symptoms, antithrombotic therapy, and Khorana score. The Khorana score is a validated tool that stratifies patients with cancer according to their risk of thrombosis based on tumor type, hematologic parameters, and BMI.^9^

Categorical variables were compared using χ^2^ or Fisher exact tests, and continuous variables using Student *t*-tests or Wilcoxon rank-sum tests as appropriate. Survival outcomes were estimated using Kaplan-Meier methods and compared with log-rank tests. Median follow-up was calculated using the reverse Kaplan-Meier method.^10^ Statistical analyses were performed using R version 4.3.1.

In this study, baseline characteristics were generally well balanced between the 2 cohorts, including age, sex, cardiovascular risk factors, BMI, smoking history, prior thrombotic events, cancer type, and metastatic status (63% in the COVID-19 cohort vs 59.3% in the non-COVID-19 cohort; *P* = .6). The distribution of Khorana scores and hospitalization rates was also similar (Table 1).

**Table 1.** Baseline characteristics.

Variable	Non-COVID-19 cohort	COVID-19 cohort	Statistical test	*P*	Missing data
**Number of patients**	214	119			
**Median age, year**	68.4 (60.9-77.4)	68.8 (62-78.5)	Rank sum	.5	0 (0%)
**Gender**					1 (0.3%)
** Female**	97 (45.5%)	51 (42.9%)	Chi square	.7	
** Male**	116 (54.5%)	68 (57.1%)			
**Median body mass index**	24 (21-26)	24 (22-27)	Rank sum	.7	14 (4.2%)
**Tobacco consumption**					4 (1.2%)
** Active**	70 (32.7%)	41 (35.7%)	Chi square	.7	
** Nonactive**	144 (67.3%)	74 (64.3%)			
**High blood pressure**	79 (36.9%)	48 (40.3%)	Chi square	.6	0 (0%)
**Dyslipidemia**	39 (18.2%)	24 (20.2%)	Chi square	.7	0 (0%)
**Diabetes**	44 (20.6%)	18 (15.1%)	Chi square	.3	0 (0%)
**History of venous thrombosis**	31 (14.5%)	17 (14.3%)	Chi square	1	0 (0%)
**History of arterial thrombosis**	23 (10.7%)	18 (15.1%)	Chi square	.3	0 (0%)
**Antithrombotic drug**					0 (0%)
** None**	134 (62.6%)	52 (43.7%)	Fisher’s exact	<.001	
** Antiplatelet agent**	38 (17.8%)	17 (14.3%)			
** Anticoagulant**	40 (18.7%)	45 (37.8%)			
** Both**	2 (0.9%)	5 (4.2%)			
**Metastatic/disseminated**	127 (59.3%)	75 (63%)	Chi square	.6	0 (0%)
**Palliative care (no active oncologic treatment)**	21 (9.8%)	48 (40.3%)	Chi square	<.001	0 (0%)
**ECOG performance status**					15 (4.5%)
** 0-1**	148 (72.5%)	34 (29.8%)	Chi square	<.001	
** 2-3-4**	56 (27.5%)	80 (70.2%)			
**Khorana score**					0 (0%)
** <2**	146 (68.2%)	75 (63%)	Fisher’s exact	.6	
** 2**	51 (23.8%)	30 (25.2%)			
** 3**	15 (7%)	12 (10.1%)			
** 4**	2 (0.9%)	2 (1.7%)			
**Mean hemoglobin (SD)**	12.3 g dL^–1^ (1.9)	10.7 g dL^–1^ (1.9)	*t*-Test	<.001	2 (0.6%)
**Median leucocytes (interquartile range)**	7085 µL^–1^ (5445-9335)	6600 µL^–1^ (3960-11 200)	Rank sum	.3	2 (0.6%)
**Median platelets (interquartile range)**	243 500 µL^–1^ (174 500-318 750)	184 000 µL^–1^ (105 000-248 000)	Rank sum	<.001	2 (0.6%)
**Hospitalized**	172 (80.4%)	101 (84.9%)	Chi square	.4	0 (0%)
**Cancer histology**					0 (0%)
** Colorectal**	17 (7.9%)	10 (8.4%)	Chi square	.5	
** Stomach**	3 (1.4%)	1 (0.8%)			
** Genitourinary**	9 (4.2%)	5 (4.2%)			
** Gynecologic**	11 (5.1%)	6 (5%)			
** Hematologic**	22 (10.3%)	14 (11.8%)			
** Head and neck**	13 (6.1%)	7 (5.9%)			
** Pancreas**	17 (7.9%)	9 (7.6%)			
** Lung**	61 (28.5%)	33 (27.7%)			
** Prostate**	11 (5.1%)	6 (5%)			
** Breast**	16 (7.5%)	8 (6.7%)			
** Other**	34 (15.9%)	20 (16.8%)			

Abbreviation: ECOG = Eastern Cooperative Oncology Group.

However, patients with COVID-19 had more advanced disease and poorer general condition. A significantly higher proportion were treated with palliative intent (40.3% vs 9.8%; *P* < .001), and fewer had an ECOG performance status of 0-1 (29.8% vs 72.5%; *P* < .001). Primarily due to poorer ECOG performance status, patients in the COVID-19 cohort more frequently received prophylactic antithrombotic therapy (56.3% vs 37.4%; *P* < .001). Ninety-four patients (78.9%) in the COVID-19 cohort presented with COVID-19–related symptoms.

After a median follow-up of 32.7 months (95% CI = 26.7 to 40.9), 29 thrombotic events were observed in the overall population, including 25 venous and 4 arterial events (a detailed description of thrombotic events is provided in Table S1). The cumulative incidence of thrombotic events was similar between cohorts (8.4% in the COVID-19 cohort vs 8.8% in the non-COVID-19 cohort; *P* = .4). Freedom from venous thromboembolism at 6 and 12 months was 90% and 90% in the COVID-19 cohort compared with 93% and 92% in the non-COVID-19 cohort (*P* = .3). Arterial events were rare, with freedom from arterial thrombosis of 99% at both time points in both groups. Early venous-thromboembolic (VTE) rates at 1 and 2 months were numerically higher in the COVID-19 cohort (7% and 9%) than in the non-COVID-19 cohort (2% and 6%), although these differences were not statistically significant (*P* = .4). Consistently, Fine-Gray competing-risk analyses accounting for the risk of death showed no significant difference between cohorts for overall thrombotic events (sHR 1.04, 95% CI = 0.49 to 2.22; *P* = .9), venous thromboembolic events (subdistribution hazard ratio [sHR] 1.12, 95% CI = 0.50 to 2.51; *P* = .8), or arterial thrombotic events (sHR 0.62, 95% CI = 0.07 to 5.87; *P* = .7).

In univariate analyses, the Khorana score was the only significant factor associated with thrombotic risk. Patients with scores >1 had higher cumulative event rates compared with those with lower scores (14.3% vs 5.9%; log-rank *P* = .005). Other variables were not associated with thrombotic risk in the overall cohort (Table S2).

Subgroup analyses revealed a potential differential effect of COVID-19 according to antithrombotic therapy. Among patients not receiving antithrombotic treatment, COVID-19 was associated with a significantly higher 6-month cumulative incidence of thrombosis (14% [95% CI = 3 to 25] vs 5% [95% CI = 1 to 9]; *P* = .038, Figure 1, A). In this subgroup, a Khorana score ≥2 was also associated with increased thrombotic risk (15% vs 5%; *P* = .044, Figure 1, B). A log-rank test showed a significant difference in event-free survival across the 4 groups. (pre-COVID/COVID/Khorana < 2/Khorana ≥ 2) (χ^2^ = 14.1, df = 3, *P* = .003). The association between COVID-19 and thrombotic risk was no longer statistically significant after accounting for the competing risk of death using the Fine-Gray model (sHR 2.42, 95% CI = 0.82 to 7.12; *P* = .10). Similarly, the association between the Khorana score and thrombotic risk was not statistically significant (sHR 2.79 95% CI = 0.94 to 8.28; *P* = .06).

**Figure 1.**
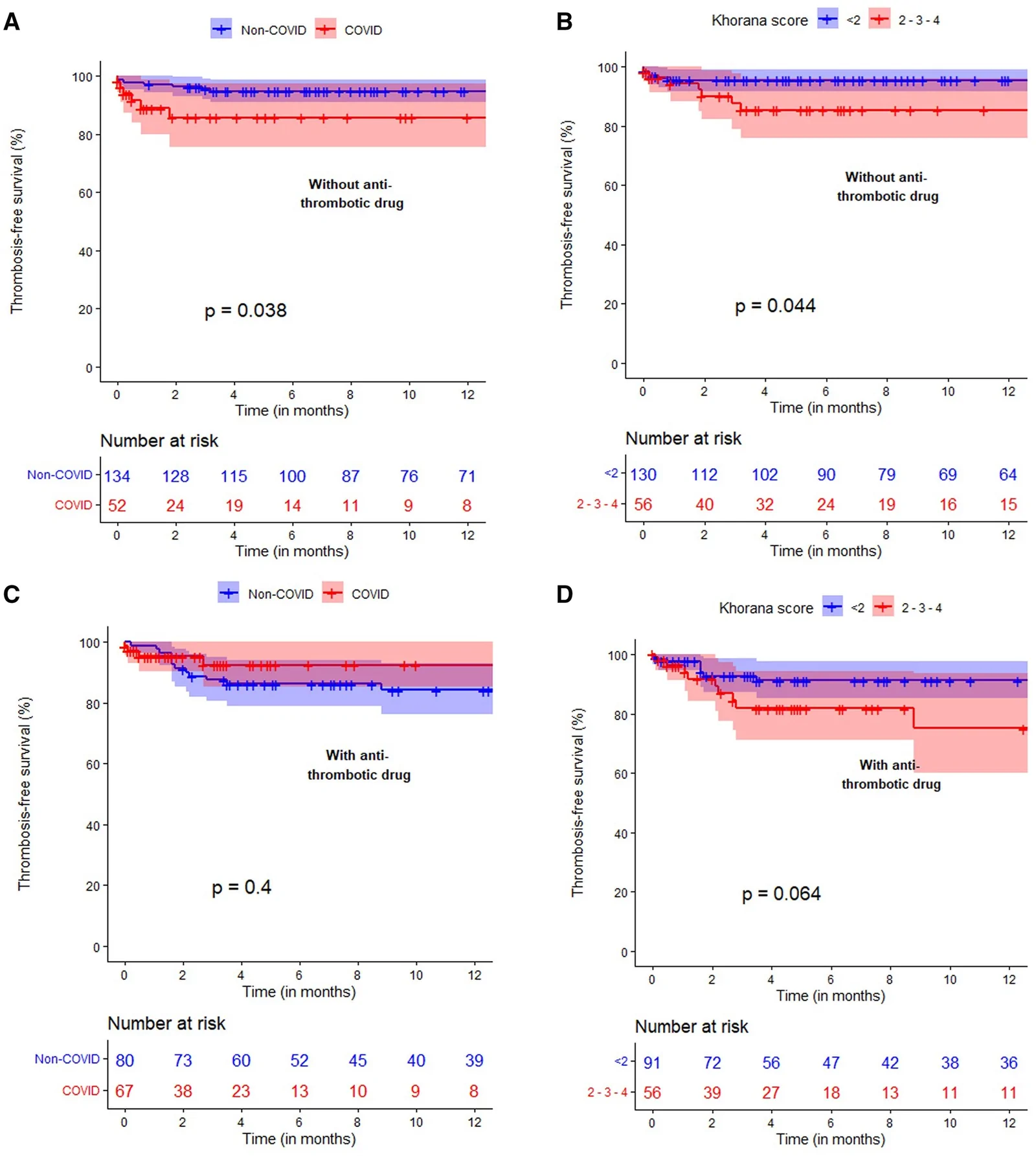
Thrombosis-free survival (A) in patients without antithrombotic drug, with/without COVID, (B) in patients without antithrombotic drug, as a function of Khorana score, (C) in patients with antithrombotic drug, with/without COVID, (D) in patients with antithrombotic drug, as a function of Khorana score (log-rank).

Among patients receiving antithrombotic therapy (*n* = 80 and 67, respectively), no significant difference in thrombotic event rates was observed between cohorts (8% vs 14%; *P* = .4, Figure 1, C), as well as for the Khorana score (*P* = .06, Figure 1, D). No significant association was observed also in the Fine-Gray competing-risk analysis for COVID-19 status (sHR 0.43, 95% CI = 0.14 to 1.33; *P* = .14). Similarly, the association between a Khorana score >1 and thrombotic risk was not statistically significant (sHR 2.19 95% CI = 0.85 to 5.84; *P* = .11).

We also performed multivariable regression analyses adjusting for COVID-19 status, ECOG performance status, treatment intent (palliative vs nonpalliative), cancer type, Khorana score, and antithrombotic treatment. When death was considered as a competing event, the multivariable Fine-Gray analysis identified COVID-19 status (sHR 1.74, 95% CI = 1.23 to 2.45; *P* = .002) and palliative treatment intent (sHR 2.41, 95% CI = 1.42 to 4.09; *P* = .0012) as independently associated with a higher cumulative incidence of thrombotic events (Table S3).

Overall survival differed markedly between groups. A total of 71 deaths occurred in the COVID-19 cohort and 125 in the non-COVID-19 cohort. Median OS was 4.1 months (interquartile range [IQR], 3.1-7.9) in the COVID-19 cohort compared with 18.7 months (IQR, 15-26) in the non-COVID-19 cohort (log-rank *P* < .001, Figure 2). Estimated 12-month OS rates were 36% (95% CI = 28 to 48) and 63% (95% CI = 57 to 70), respectively.

**Figure 2.**
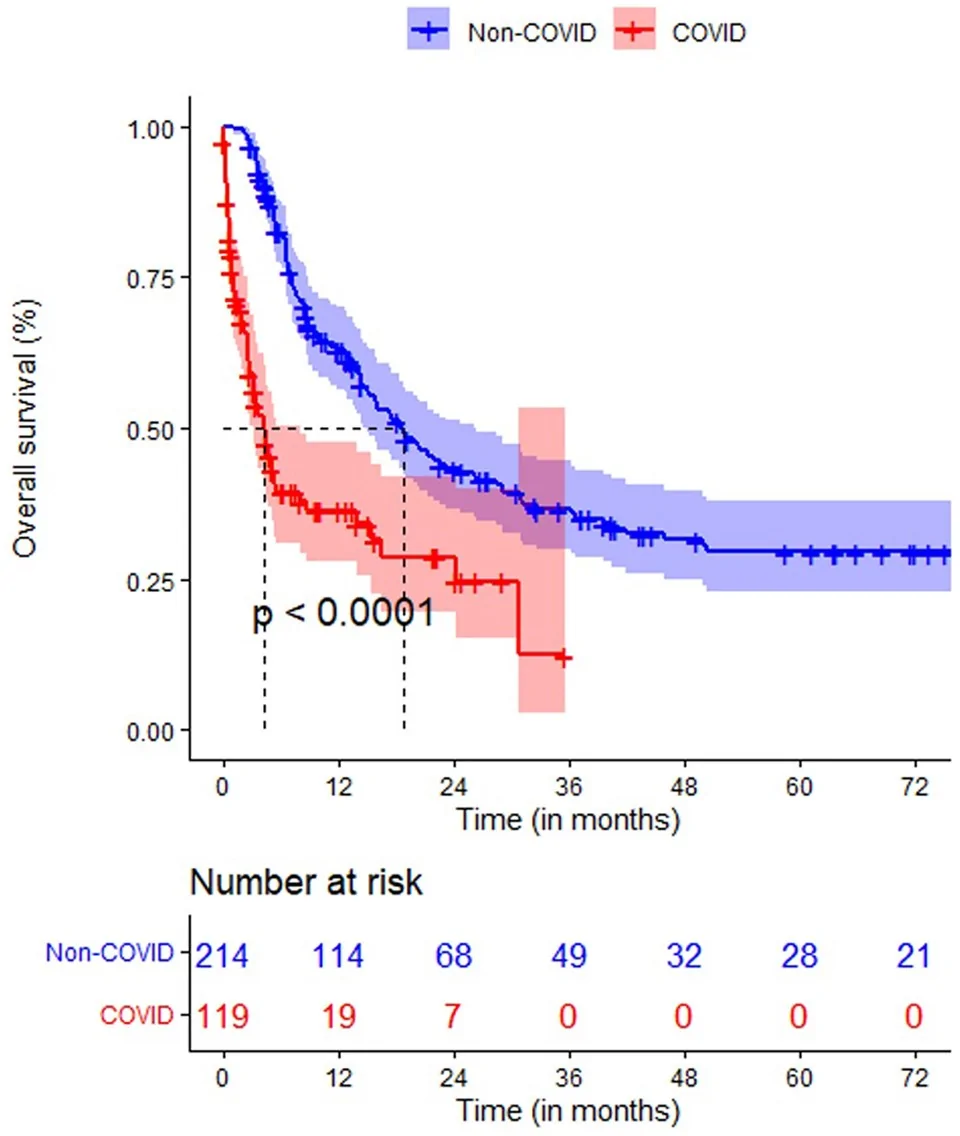
Overall survival.

Among deaths in the COVID-19 cohort, the majority were attributable to cancer progression (70.4%), followed by COVID-19–related respiratory disease (15.5%). Other causes were unknown. Palliative treatment intent was the only variable significantly associated with mortality (*P* < .001).

In this matched cohort study, the overall incidence of thrombotic events in patients with active cancer was approximately 8%-9%, consistent with prior reports in similar populations.^11^ COVID-19 was not associated with an increased risk of thrombosis in the overall cohort but was associated with higher risk in patients not receiving antithrombotic therapy. Interestingly, after adjustment for clinically relevant covariates and accounting for death as a competing event, COVID-19 status and palliative treatment intent remained independently associated with a higher cumulative incidence of thrombotic events. Although several studies have described thrombotic complications in patients with cancer and COVID-19, they primarily compared patients with and without cancer among COVID-19 cohorts or focused exclusively on patients with cancer and COVID-19.^12^^,^^13^ Our study extends this literature by directly comparing matched hospitalized patients with cancer with and without COVID-19.

These findings suggest that SARS-CoV-2 infection may exacerbate thrombotic risk in patients with cancer maybe particularly in the absence of thromboprophylaxis, while standard prophylactic strategies may mitigate this effect. During the study period, antithrombotic management was not systematically modified in response to COVID-19 but followed standard oncology practice, consistent with European guidelines recommending thromboprophylaxis based on cancer-related risk factors rather than COVID-19 status alone.^14^ Randomized trials in noncancer populations have shown that therapeutic-dose anticoagulation may improve outcomes in selected patients with COVID-19.^15^^,^^16^ However, these studies did not specifically evaluate patients with active cancer.

The Khorana score was confirmed as a significant predictor of thrombotic events, in line with its established role in cancer-associated thrombosis.^9^ Other clinical variables, including performance status, were not associated with thrombotic risk, supporting the importance of tumor-related and biological factors. Patients with COVID-19 in this study had more advanced disease and poorer performance status, which likely contributed to their worse survival outcomes. Similar observations have been reported in other cohorts,^17^ although patients receiving palliative care were not included. Advanced cancer is associated with immune dysfunction, including lymphopenia and T-cell exhaustion, which may increase susceptibility to infections.^18^^,^^19^

This study has several limitations. First, the retrospective design, the relatively modest sample size, and the low number of thrombotic events make it difficult to extrapolate our findings to broader populations. Second, venous and arterial events were analyzed together to increase statistical power, and different antithrombotic agents were grouped despite potential differences in mechanism, although antiplatelet agents may reduce VTE risk in some settings.^20^ Third, the matched cohort and the COVID-19 cohort were recruited during different time periods (2011-2017 and 2020-2022, respectively). Changes in cancer treatment, diagnostic strategies, and thromboprophylaxis practices over time may have acted as confounding factors and could partly explain the differences observed between the 2 cohorts. Fourth, baseline differences in cancer treatment, vaccination status, the dominant SARS-CoV-2 variant, and COVID-19 treatments between the 2 cohorts may also have acted as confounding factors, partially explaining the observed differences. In addition, despite matching, some baseline imbalances remained between the cohorts, particularly in palliative intent, ECOG performance status, hemoglobin, platelet count, and antithrombotic use. Furthermore, selection bias related to the inclusion of more severely ill hospitalized patients and detection bias due to more frequent imaging in patients with COVID-19 cannot be excluded. Fifth, the reference date in the control cohort was defined as the date of the first documented medical assessment associated with an available blood test, as laboratory values were required to calculate the Khorana score. This may have introduced some temporal heterogeneity compared with the study cohort. However, the proportion of patients with metastatic disease was ultimately similar between the 2 groups. Sixth, the higher incidence of thrombotic events was observed only in a subgroup analysis and should be confirmed in further prospective studies.

In conclusion, these findings suggest that COVID-19 may increase thrombotic risk in cancer patients and COVID-19 occurs more frequently in patients with poorer clinical status. Our findings suggest that the role of anticoagulation in this population deserves further investigation. These results are exploratory and require confirmation in larger prospective studies.

## Supplementary Material

pkag110_Supplementary_Data

## Data Availability

The data will be shared upon reasonable request to the corresponding author.
