# Supplementary material for: COVID-19 and thrombotic risk in patients with active cancer: a matched cohort study
Source: JNCI Cancer Spectr. 2026 Oct 12;10(5):pkag110. doi: 10.1093/jncics/pkag110 (PMC13713195; doi:10.1093/jncics/pkag110)
Supplement: Supplementary file 1 [file pkag110_supplementary_data.zip › Table S1.docx]

**Table S1. Detailed description of thrombotic events**

|  | **Non-COVID-19 cohort** | **COVID-19 cohort** | **1-year FVTE*** | **Log-rank** |
| --- | --- | --- | --- | --- |
| **Venous thromboses** | Pulmonary embolism (n=6)  Deep venous thrombosis  (n=8)  Pulmonary embolism and Deep venous thrombosis (n=1)  Superficial venous thrombosis (n=1) | Pulmonary embolism (n=8)  Pulmonary embolism + Deep venous thrombosis (n=1) | Non-COVID-19 cohort: 92% [95%CI 88-96]  vs  COVID-19 cohort: 90% [95%CI 84-97] | p=0.3 |
|  | **Non-COVID-19 cohort** | **COVID-19 cohort** | **1-year FATE**** | **Log-rank** |
| **Arterial thromboses** | Ischemic stroke (n=1)  Myocardial infarction (n=2) | Ischemic stroke (n=1) | Non-COVID-19 cohort: 99% [95%CI 97-100]   vs  COVID-19 cohort: 99% [95%CI 98-100] | p=0.8 |

*FVTE: freedom-free survival from venous thrombotic events
**FATE: freedom-free survival from arterial thrombotic events
