# Supplementary material for: COVID-19 and thrombotic risk in patients with active cancer: a matched cohort study
Source: JNCI Cancer Spectr. 2026 Oct 12;10(5):pkag110. doi: 10.1093/jncics/pkag110 (PMC13713195; doi:10.1093/jncics/pkag110)
Supplement: Supplementary file 1 [file pkag110_supplementary_data.zip › Table S2.docx]

**Table S2. Predictive factors for thrombosis (venous and arterial combined)**

| **Variable** | **Cumulative thrombosis rate** | **Hazard ratio [95%CI]** | **p-value** |
| --- | --- | --- | --- |
| **Median age** |  | 1.01 [0.98-1.04] | 0.6 (Cox) |
| **Median body mass index** |  | 0.97 [0.89-1.05] | 0.5 (Cox) |
| **Non-COVID-19 cohort COVID-19 cohort** | 8.9% 8.4% |  | 0.4 (Log-Rank) |
| **Gender** Female Male | 11.5% 6.5% |  | 0.13 (Log-Rank) |
| **Tobacco consumption** Active Non active | 11.7% 7.3% |  | 0.2 (Log-Rank) |
| **High blood pressure** Yes No | 7% 9.7% |  | 0.4 (Log-Rank) |
| **Dyslipidemia** Yes No | 7.9% 8.9% |  | 0.8 (Log-Rank) |
| **Diabetes** Yes No | 8.1% 8.9% |  | 0.7 (Log-Rank) |
| **History of venous thrombosis** Yes No | 14.6% 7.7% |  | 0.09 (Log-Rank) |
| **History of arterial thrombosis** Yes No | 4.9% 9.2% |  | 0.4 (Log-Rank) |
| **Anti-thrombotic drug** None antiplatelet agent anticoagulant agent antiplatelet and anticoagulant agents | 7% 7.3% 12.9% 14.3% |  | 0.17 (Log-Rank) |
| **Palliative care (no active oncologic treatment)** Yes No | 5.8% 9.5% |  | 0.9 (Log-Rank) |
| **ECOG* performance status** 0-1 2-3-4 | 8.8% 9.6% |  | 0.4 (Log-Rank) |
| **Khorana score** 0-1 2-3-4 | 5.9% 14.3% |  | 0.005 (Log-Rank) |
| **Infection symptoms** Yes No | 4.7% 9.7% |  | 0.5 (Log-Rank) |
| **Hospitalized** Yes No | 8% 11.7% |  | 0.5 (Log-Rank) |

*Eastern Cooperative Group
