# Supplementary material for: COVID-19 and thrombotic risk in patients with active cancer: a matched cohort study
Source: JNCI Cancer Spectr. 2026 Oct 12;10(5):pkag110. doi: 10.1093/jncics/pkag110 (PMC13713195; doi:10.1093/jncics/pkag110)
Supplement: Supplementary file 1 [file pkag110_supplementary_data.zip › Table S3.docx]

**Table S3 : Univariable and multivariable Fine-Gray competing risk analysis of both venous and arterial thrombotic events (death as a competing event)**

|  | **Univariable analysis** | | **Multivariable analysis** | |
| --- | --- | --- | --- | --- |
| **Variables** | **HR [CI95%]** | **Pvalue** | **Subdistribution HR [CI95%]** | **Pvalue** |
| Cancer type : High risk vs Standard risk* | 0.95 [0.76; 1.20] | 0.6884 | 0.93 [0.70; 1.24] | 0.6243 |
| Cancer type : Very high risk vs Standard risk** | 0.67 [0.37; 1.22] | 0.1866 | 0.51 [0.24; 1.08] | 0.0795 |
| Palliative care: Yes vs No | 3.14 [1.93; 5.10] | <0.001 | 2.41 [1.42; 4.09] | 0.0012 |
| Cohorte : COVID vs Non-COVID | 2.21 [1.61; 3.04] | <0.001 | 1.74 [1.23; 2.45] | 0.0016 |
| Khorana score : 2-3-4 vs 0-1 | 1.13 [0.85; 1.48] | 0.4023 | 1.21 [0.85; 1.71] | 0.2844 |
| ECOG performance status :_2-3-4 vs 0-1 | 1.69 [1.30; 2.20] | <0.001 | 1.26 [0.90; 1.75] | 0.1731 |
| Antithrombotic treatment: Yes vs No | 1.07 [0.84; 1.36] | 0.5677 | 0.89 [0.68; 1.17] | 0.4095 |

ECOG : Eastern Cooperative Oncology Group
* and **
Very high risk : pancreatic cancer, gastric cancer
High risk : lung cancer, lymphoma, gynecologic cancers, genitourinary cancers
Standard risk : all other cancers
